# Diaqua­bis­(4-bromo­benzoato-κ*O*)bis­(nicotinamide-κ*N* ^1^)copper(II)

**DOI:** 10.1107/S1600536811021696

**Published:** 2011-06-11

**Authors:** Hacali Necefoğlu, Füreya Elif Özbek, Vijdan Öztürk, Barış Tercan, Tuncer Hökelek

**Affiliations:** a Department of Chemistry, Kafkas University, 36100 Kars, Turkey; b Department of Physics, Karabük University, 78050, Karabük, Turkey; c Department of Physics, Hacettepe University, 06800 Beytepe, Ankara, Turkey

## Abstract

The asymmetric unit of the title mononuclear Cu^II^ complex, [Cu(C_7_H_4_BrO_2_)_2_(C_6_H_6_N_2_O)_2_(H_2_O)_2_], contains one half-mol­ecule, the Cu^II^ atom being located on an inversion center. The unit cell contains two nicotinamide (NA), two 4-bromo­benzoate (PBB) ligands and two coordinated water mol­ecules. The four O atoms in the equatorial plane around the Cu^II^ ion form a slightly distorted square-planar arrangement, while the slightly distorted octa­hedral coordination is completed by the two N atoms of the NA ligands in the axial positions. The dihedral angle between the carboxyl­ate group and the adjacent benzene ring is 22.17 (16)°, while the pyridine ring and the benzene ring are oriented at a dihedral angle of 82.80 (6)°. In the crystal, N—H⋯O, O—H⋯O and C—H⋯O hydrogen bonds link the mol­ecules into a three-dimensional network. A weak C—H⋯π inter­action is also observed.

## Related literature

For literature on niacin, see: Krishnamachari (1974 ▶). For infomation on the nicotinic acid derivative *N*,*N*-diethyl­nicotinamide, see: Bigoli *et al.* (1972 ▶). For related structures, see: Hökelek *et al.* (1996 ▶, 2009*a* ▶,*b* ▶); Hökelek & Necefoğlu (1998 ▶, 2007 ▶); Necefoğlu *et al.* (2011 ▶). For bond-length data, see: Allen *et al.* (1987 ▶).
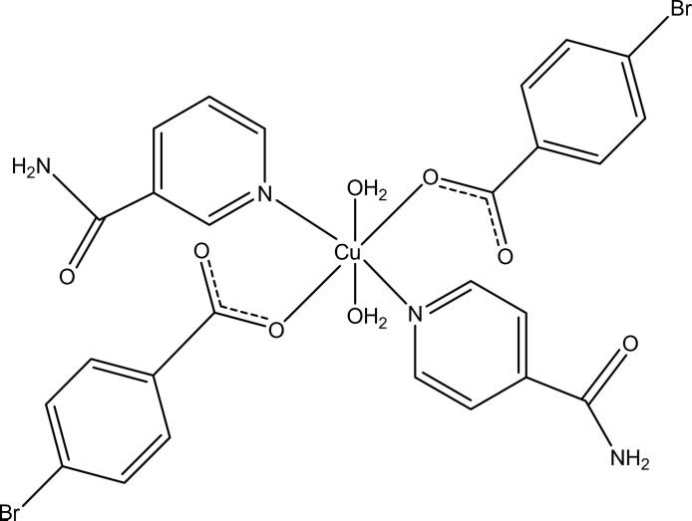


## Experimental

### 

#### Crystal data

[Cu(C_7_H_4_BrO_2_)_2_(C_6_H_6_N_2_O)_2_(H_2_O)_2_]
*M* *_r_* = 743.84
Triclinic, 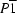

*a* = 7.7072 (3) Å
*b* = 9.7536 (5) Å
*c* = 9.8471 (4) Å
α = 76.273 (2)°
β = 74.240 (2)°
γ = 85.024 (3)°
*V* = 691.86 (5) Å^3^
*Z* = 1
Mo *K*α radiation
μ = 3.73 mm^−1^
*T* = 100 K
0.41 × 0.38 × 0.35 mm

#### Data collection

Bruker Kappa APEXII CCD area-detector diffractometer
Absorption correction: multi-scan (*SADABS*; Bruker, 2005 ▶) *T*_min_ = 0.236, *T*_max_ = 0.271
11758 measured reflections
3515 independent reflections
3172 reflections with *I* > 2σ(*I*)
*R*_int_ = 0.057

#### Refinement

*R*[*F* ^2^ > 2σ(*F* ^2^)] = 0.039
*wR*(*F* ^2^) = 0.108
*S* = 1.13
3515 reflections
203 parameters
H atoms treated by a mixture of independent and constrained refinement
Δρ_max_ = 0.86 e Å^−3^
Δρ_min_ = −1.29 e Å^−3^

### 

Data collection: *APEX2* (Bruker, 2007 ▶); cell refinement: *SAINT* (Bruker, 2007 ▶); data reduction: *SAINT*; program(s) used to solve structure: *SHELXS97* (Sheldrick, 2008 ▶); program(s) used to refine structure: *SHELXL97* (Sheldrick, 2008 ▶); molecular graphics: *Mercury* (Macrae *et al.*, 2006 ▶); software used to prepare material for publication: *WinGX* publication routines (Farrugia, 1999 ▶) and *PLATON* (Spek, 2009 ▶).

## Supplementary Material

Crystal structure: contains datablock(s) I, global. DOI: 10.1107/S1600536811021696/su2279sup1.cif

Structure factors: contains datablock(s) I. DOI: 10.1107/S1600536811021696/su2279Isup2.hkl

Additional supplementary materials:  crystallographic information; 3D view; checkCIF report

## Figures and Tables

**Table 1:** Hydrogen-bond geometry (Å, °) *Cg* is the centroid of the N1/C8–C12 pyridine ring.

*D*—H⋯*A*	*D*—H	H⋯*A*	*D*⋯*A*	*D*—H⋯*A*
N2—H21⋯O2^i^	0.85 (3)	2.06 (3)	2.831 (2)	151 (3)
N2—H22⋯O3^ii^	0.88 (3)	2.03 (3)	2.893 (3)	166 (3)
O4—H41⋯O2^iii^	0.87 (4)	1.86 (4)	2.718 (2)	167 (4)
O4—H42⋯O3^iv^	0.78 (4)	2.17 (4)	2.911 (2)	159 (4)
C6—H6⋯O2^v^	0.95	2.43	3.377 (3)	172
C4—H4⋯*Cg*^vi^	0.95	2.63	3.581 (3)	176

Symmetry codes: (i) 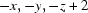; (ii) 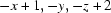; (iii) 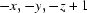; (iv) 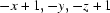; (v) 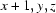; (vi) 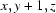.

**Fig. 1.**
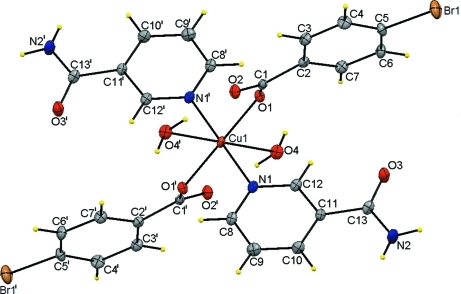
The molecular structure of the title molecule with the atom-numbering scheme. Displacement ellipsoids are drawn at the 50% probability level [symmetry code: (') -x, -y, -z+1].

## supplementary crystallographic information

### Comment

As a part of our ongoing investigations of transition metal complexes of nicotinamide (NA), one form of niacin (Krishnamachari, 1974), and/or the nicotinic acid derivative *N*,*N*-diethylnicotinamide (DENA), an important respiratory stimulant (Bigoli *et al.*, 1972), the title compound was synthesized and its crystal structure is reported herein.

The asymmetric unit of the title mononuclear Cu^II^complex, (Fig. 1), contains one-half molecule. It consists of two nicotinamide (NA), two 4-bromobenzoate (PBB) ligands and two coordinated water molecules, all ligands coordinating in a monodentate manner. Atom Cu1 is located on an inversion center. The crystal structures of similar complexes of Cu^II^, Co^II^, Ni^II^, Mn^II^ and Zn^II^ ions, [Cu(C_7_H_5_O_2_)_2_(C_10_H_14_N_2_O)_2_] (Hökelek *et al.*, 1996), [Co(C_6_H_6_N_2_O)_2_(C_7_H_4_NO_4_)_2_(H_2_O)_2_] (Hökelek & Necefoğlu, 1998), [Co(C_9_H_9_O_2_)_2_(C_10_H_14_N_2_O)_2_(H_2_O)_2_] (Necefoğlu *et al.*, 2011), [Ni(C_7_H_4_ClO_2_)_2_(C_6_H_6_N_2_O)_2_(H_2_O)_2_] (Hökelek *et al.*, 2009*a*), [Mn(C_9_H_10_NO_2_)_2_(H_2_O)_4_].2H_2_O (Hökelek & Necefoğlu, 2007) and [Zn(C_7_H_4_BrO_2_)_2_(C_6_H_6_N_2_O)_2_(H_2_O)_2_] (Hökelek *et al.*, 2009*b*) have also been reported. In the copper(II) complex mentioned above the two benzoate ions coordinate to the Cu^II^ atom as bidentate ligands, while in the other structures all the ligands coordinate in a monodentate manner.

In the title complex, the four symmetry related O atoms (O1, O1', O4 and O4') in the equatorial plane around the Cu^II^ ion form a slightly distorted square-planar arrangement, while the slightly distorted octahedral coordination is completed by the two symmetry related N atoms of the NA ligands (N1 and N1') in the axial positions. The near equalities of the C1—O1 [1.272 (3) Å] and C1—O2 [1.246 (3) Å] bonds in the carboxylate group indicate delocalized bonding arrangement, rather than localized single and double bonds. The Cu—O bond lengths are 1.9756 (16) Å (for benzoate oxygens) and 2.4199 (16) Å (for water oxygens), and the Cu—N bond length is 2.0116 (16) Å, close to standard values (Allen *et al.*, 1987). The Cu atom is displaced out of the mean-plane of the carboxylate group (O1/C1/O2) by -0.5279 (1) Å. The dihedral angle between the planar carboxylate group and the adjacent benzene ring A (C2—C7) is 22.17 (16)°. The benzene A (C2—C7) and the pyridine B (N1/C8—C12) rings are oriented at a dihedral angle of A/B = 82.80 (6)°.

In the crystal, intermolecular N—H···O, O—H···O and C—H···O hydrogen bonds link the molecules into a three-dimensional network (Table 1). There also exists a weak C-H···π interaction (Table 1).

### Experimental

The title compound was prepared by the reaction of CuSO_4_.5H_2_O (1.23 g, 5 mmol) in H_2_O (20 ml) and NA (1.22 g, 10 mmol) in H_2_O (20 ml) with sodium 4-bromobenzoate (2.23 g, 10 mmol) in H_2_O (100 ml) at room temperature. The mixture was filtered and set aside to crystallize at ambient temperature for three weeks, giving blue single crystals.

### Refinement

Atoms H21 and H22 (for NH_2_) and H41 and H42 (for H_2_O) were located in a difference Fourier map and were freely refined. The C-bound H-atoms were positioned geometrically with C—H = 0.95 Å for aromatic H-atoms, and constrained to ride on their parent atoms, with *U*_iso_(H) = *1.2U*_eq_(C).

### Crystal data

**Table:**

[Cu(C_7_H_4_BrO_2_)_2_(C_6_H_6_N_2_O)_2_(H_2_O)_2_]	*Z* = 1
*M**_r_* = 743.84	*F*(000) = 371
Triclinic, *P*1	*D*_x_ = 1.785 Mg m^−^^3^
Hall symbol: -P 1	Mo *K*α radiation, λ = 0.71073 Å
*a* = 7.7072 (3) Å	Cell parameters from 7694 reflections
*b* = 9.7536 (5) Å	θ = 2.2–28.7°
*c* = 9.8471 (4) Å	µ = 3.73 mm^−^^1^
α = 76.273 (2)°	*T* = 100 K
β = 74.240 (2)°	Block, blue
γ = 85.024 (3)°	0.41 × 0.38 × 0.35 mm
*V* = 691.86 (5) Å^3^	

### Data collection

**Table:**

Bruker Kappa APEXII CCD area-detector diffractometer	3515 independent reflections
Radiation source: fine-focus sealed tube	3172 reflections with *I* > 2σ(*I*)
graphite	*R*_int_ = 0.057
φ and ω scans	θ_max_ = 28.7°, θ_min_ = 2.2°
Absorption correction: multi-scan (*SADABS*; Bruker, 2005)	*h* = −10→10
*T*_min_ = 0.236, *T*_max_ = 0.271	*k* = −12→13
11758 measured reflections	*l* = −13→13

### Refinement

**Table:**

Refinement on *F*^2^	Primary atom site location: structure-invariant direct methods
Least-squares matrix: full	Secondary atom site location: difference Fourier map
*R*[*F*^2^ > 2σ(*F*^2^)] = 0.039	Hydrogen site location: inferred from neighbouring sites
*wR*(*F*^2^) = 0.108	H atoms treated by a mixture of independent and constrained refinement
*S* = 1.13	*w* = 1/[σ^2^(*F*_o_^2^) + (0.0563*P*)^2^ + 0.169*P*] where *P* = (*F*_o_^2^ + 2*F*_c_^2^)/3
3515 reflections	(Δ/σ)_max_ = 0.001
203 parameters	Δρ_max_ = 0.86 e Å^−^^3^
0 restraints	Δρ_min_ = −1.29 e Å^−^^3^

### Special details

**Table:**

Geometry. All esds (except the esd in the dihedral angle between two l.s. planes) are estimated using the full covariance matrix. The cell esds are taken into account individually in the estimation of esds in distances, angles and torsion angles; correlations between esds in cell parameters are only used when they are defined by crystal symmetry. An approximate (isotropic) treatment of cell esds is used for estimating esds involving l.s. planes.
Refinement. Refinement of F^2^ against ALL reflections. The weighted R-factor wR and goodness of fit S are based on F^2^, conventional R-factors R are based on F, with F set to zero for negative F^2^. The threshold expression of F^2^ > 2sigma(F^2^) is used only for calculating R-factors(gt) etc. and is not relevant to the choice of reflections for refinement. R-factors based on F^2^ are statistically about twice as large as those based on F, and R- factors based on ALL data will be even larger.

### Fractional atomic coordinates and isotropic or equivalent isotropic displacement parameters (Å^2^)

**Table:**

	*x*	*y*	*z*	*U*_iso_*/*U*_eq_	
Br1	0.50803 (3)	0.60173 (3)	0.78887 (3)	0.02585 (11)	
Cu1	0.0000	0.0000	0.5000	0.00979 (12)	
O1	0.1042 (2)	0.17464 (16)	0.50913 (16)	0.0123 (3)	
O2	−0.1351 (2)	0.26522 (18)	0.64918 (17)	0.0160 (3)	
O3	0.4297 (2)	−0.00851 (18)	0.84131 (16)	0.0182 (4)	
O4	0.3099 (2)	−0.0838 (2)	0.43625 (18)	0.0172 (4)	
H41	0.271 (5)	−0.146 (4)	0.402 (4)	0.036 (9)*	
H42	0.383 (5)	−0.042 (4)	0.371 (4)	0.043 (11)*	
N1	−0.0003 (2)	−0.08494 (19)	0.70725 (18)	0.0111 (4)	
N2	0.3330 (3)	−0.1428 (2)	1.0661 (2)	0.0176 (4)	
H21	0.251 (4)	−0.189 (3)	1.134 (3)	0.028 (10)*	
H22	0.417 (4)	−0.110 (3)	1.094 (3)	0.016 (7)*	
C1	0.0294 (3)	0.2585 (2)	0.5904 (2)	0.0103 (4)	
C2	0.1508 (3)	0.3509 (2)	0.6240 (2)	0.0116 (4)	
C3	0.0789 (3)	0.4675 (2)	0.6809 (2)	0.0149 (4)	
H3	−0.0442	0.4943	0.6883	0.018*	
C4	0.1860 (3)	0.5448 (3)	0.7267 (3)	0.0174 (5)	
H4	0.1375	0.6251	0.7644	0.021*	
C5	0.3644 (3)	0.5032 (2)	0.7167 (2)	0.0145 (4)	
C6	0.4403 (3)	0.3898 (2)	0.6570 (2)	0.0158 (5)	
H6	0.5638	0.3638	0.6490	0.019*	
C7	0.3328 (3)	0.3151 (2)	0.6094 (2)	0.0144 (4)	
H7	0.3837	0.2384	0.5663	0.017*	
C8	−0.1393 (3)	−0.1592 (2)	0.8002 (2)	0.0132 (4)	
H8	−0.2407	−0.1721	0.7675	0.016*	
C9	−0.1386 (3)	−0.2178 (3)	0.9430 (2)	0.0170 (5)	
H9	−0.2382	−0.2703	1.0072	0.020*	
C10	0.0077 (3)	−0.1992 (2)	0.9903 (2)	0.0150 (4)	
H10	0.0101	−0.2388	1.0877	0.018*	
C11	0.1524 (3)	−0.1221 (2)	0.8948 (2)	0.0109 (4)	
C12	0.1421 (3)	−0.0671 (2)	0.7527 (2)	0.0122 (4)	
H12	0.2405	−0.0151	0.6859	0.015*	
C13	0.3162 (3)	−0.0880 (2)	0.9325 (2)	0.0137 (5)	

### Atomic displacement parameters (Å^2^)

**Table:**

	*U*^11^	*U*^22^	*U*^33^	*U*^12^	*U*^13^	*U*^23^
Br1	0.02453 (18)	0.03006 (18)	0.03230 (18)	−0.00115 (12)	−0.01163 (13)	−0.01980 (13)
Cu1	0.0109 (2)	0.0129 (2)	0.00714 (19)	0.00162 (14)	−0.00356 (14)	−0.00453 (14)
O1	0.0122 (7)	0.0149 (8)	0.0112 (7)	−0.0005 (6)	−0.0023 (6)	−0.0064 (6)
O2	0.0104 (8)	0.0214 (9)	0.0164 (8)	0.0013 (6)	−0.0016 (6)	−0.0073 (6)
O3	0.0165 (8)	0.0292 (9)	0.0101 (7)	−0.0054 (7)	−0.0030 (6)	−0.0053 (7)
O4	0.0150 (8)	0.0222 (9)	0.0147 (8)	0.0026 (7)	−0.0015 (7)	−0.0085 (7)
N1	0.0115 (9)	0.0131 (9)	0.0100 (8)	0.0037 (7)	−0.0032 (7)	−0.0062 (7)
N2	0.0172 (10)	0.0277 (11)	0.0090 (9)	−0.0027 (9)	−0.0044 (8)	−0.0043 (8)
C1	0.0091 (9)	0.0127 (10)	0.0087 (9)	−0.0004 (7)	−0.0024 (8)	−0.0014 (8)
C2	0.0141 (10)	0.0129 (10)	0.0083 (9)	0.0016 (8)	−0.0027 (8)	−0.0038 (8)
C3	0.0132 (10)	0.0162 (11)	0.0152 (10)	0.0034 (8)	−0.0025 (8)	−0.0057 (9)
C4	0.0200 (12)	0.0152 (11)	0.0189 (11)	0.0042 (9)	−0.0040 (9)	−0.0102 (9)
C5	0.0151 (11)	0.0175 (11)	0.0139 (10)	−0.0030 (8)	−0.0044 (8)	−0.0075 (8)
C6	0.0117 (10)	0.0192 (11)	0.0179 (10)	0.0029 (8)	−0.0045 (9)	−0.0070 (9)
C7	0.0157 (11)	0.0150 (11)	0.0131 (10)	0.0030 (8)	−0.0024 (8)	−0.0072 (8)
C8	0.0112 (10)	0.0169 (11)	0.0129 (10)	0.0037 (8)	−0.0030 (8)	−0.0073 (8)
C9	0.0175 (12)	0.0190 (11)	0.0129 (10)	0.0011 (9)	0.0000 (9)	−0.0055 (9)
C10	0.0153 (11)	0.0189 (11)	0.0097 (9)	0.0008 (9)	−0.0009 (8)	−0.0045 (8)
C11	0.0107 (10)	0.0140 (10)	0.0094 (9)	0.0019 (8)	−0.0025 (8)	−0.0061 (8)
C12	0.0136 (10)	0.0143 (10)	0.0100 (9)	0.0037 (8)	−0.0030 (8)	−0.0065 (8)
C13	0.0153 (11)	0.0187 (11)	0.0090 (10)	0.0043 (9)	−0.0035 (8)	−0.0082 (8)

### Geometric parameters (Å, °)

**Table:**

Br1—C5	1.895 (2)	C3—H3	0.9500
Cu1—O1	1.9756 (16)	C4—C5	1.383 (3)
Cu1—O1^i^	1.9756 (16)	C4—H4	0.9500
Cu1—N1	2.0116 (16)	C6—C5	1.386 (3)
Cu1—N1^i^	2.0116 (16)	C6—C7	1.384 (3)
Cu1—O4	2.4199 (16)	C6—H6	0.9500
Cu1—O4^i^	2.4199 (16)	C7—H7	0.9500
O1—C1	1.272 (3)	C8—N1	1.340 (3)
O2—C1	1.246 (3)	C8—H8	0.9500
O3—C13	1.238 (3)	C9—C8	1.388 (3)
O4—H41	0.87 (4)	C9—H9	0.9500
O4—H42	0.79 (4)	C10—C9	1.371 (3)
N2—C13	1.334 (3)	C10—C11	1.391 (3)
N2—H21	0.85 (3)	C10—H10	0.9500
N2—H22	0.88 (3)	C11—C12	1.396 (3)
C1—C2	1.502 (3)	C11—C13	1.493 (3)
C2—C7	1.393 (3)	C12—N1	1.331 (3)
C3—C2	1.392 (3)	C12—H12	0.9500
C3—C4	1.387 (3)		
			
O1—Cu1—O1^i^	180.0	C4—C3—C2	120.4 (2)
O1—Cu1—O4	85.01 (6)	C4—C3—H3	119.8
O1^i^—Cu1—O4	94.99 (6)	C3—C4—H4	120.5
O1—Cu1—O4^i^	94.99 (6)	C5—C4—C3	119.0 (2)
O1^i^—Cu1—O4^i^	85.01 (6)	C5—C4—H4	120.5
O1—Cu1—N1	90.51 (7)	C4—C5—Br1	119.31 (17)
O1^i^—Cu1—N1	89.49 (7)	C4—C5—C6	121.7 (2)
O1—Cu1—N1^i^	89.49 (7)	C6—C5—Br1	119.02 (17)
O1^i^—Cu1—N1^i^	90.51 (7)	C5—C6—H6	120.6
O4^i^—Cu1—O4	180.0	C7—C6—C5	118.7 (2)
N1^i^—Cu1—N1	180.0	C7—C6—H6	120.6
N1—Cu1—O4	86.82 (6)	C2—C7—H7	119.6
N1^i^—Cu1—O4	93.18 (6)	C6—C7—C2	120.8 (2)
N1—Cu1—O4^i^	93.18 (6)	C6—C7—H7	119.6
N1^i^—Cu1—O4^i^	86.82 (6)	N1—C8—C9	121.6 (2)
C1—O1—Cu1	125.94 (15)	N1—C8—H8	119.2
Cu1—O4—H41	87 (2)	C9—C8—H8	119.2
Cu1—O4—H42	122 (3)	C8—C9—H9	120.4
H41—O4—H42	105 (3)	C10—C9—C8	119.3 (2)
C8—N1—Cu1	121.83 (14)	C10—C9—H9	120.4
C12—N1—Cu1	118.84 (15)	C9—C10—C11	119.6 (2)
C12—N1—C8	119.33 (18)	C9—C10—H10	120.2
C13—N2—H21	124.3 (19)	C11—C10—H10	120.2
C13—N2—H22	118.4 (19)	C10—C11—C12	117.8 (2)
H22—N2—H21	115 (3)	C10—C11—C13	125.52 (19)
O1—C1—C2	117.12 (18)	C12—C11—C13	116.7 (2)
O2—C1—O1	125.2 (2)	N1—C12—C11	122.5 (2)
O2—C1—C2	117.63 (19)	N1—C12—H12	118.8
C3—C2—C1	119.91 (19)	C11—C12—H12	118.8
C3—C2—C7	119.4 (2)	O3—C13—N2	122.1 (2)
C7—C2—C1	120.53 (19)	O3—C13—C11	119.74 (18)
C2—C3—H3	119.8	N2—C13—C11	118.2 (2)
			
O4—Cu1—O1—C1	−150.09 (16)	C4—C3—C2—C7	−1.9 (3)
O4^i^—Cu1—O1—C1	29.91 (16)	C2—C3—C4—C5	−0.8 (3)
N1—Cu1—O1—C1	−63.33 (16)	C3—C4—C5—Br1	−176.52 (17)
N1^i^—Cu1—O1—C1	116.67 (16)	C3—C4—C5—C6	2.5 (4)
O1—Cu1—N1—C12	−42.00 (15)	C7—C6—C5—Br1	177.63 (17)
O1^i^—Cu1—N1—C12	138.00 (15)	C7—C6—C5—C4	−1.4 (4)
O1—Cu1—N1—C8	138.32 (16)	C5—C6—C7—C2	−1.4 (3)
O1^i^—Cu1—N1—C8	−41.68 (16)	C9—C8—N1—Cu1	179.86 (16)
O4—Cu1—N1—C12	42.98 (15)	C9—C8—N1—C12	0.2 (3)
O4^i^—Cu1—N1—C12	−137.02 (15)	C10—C9—C8—N1	0.1 (3)
O4—Cu1—N1—C8	−136.71 (16)	C11—C10—C9—C8	0.0 (3)
O4^i^—Cu1—N1—C8	43.29 (16)	C9—C10—C11—C12	−0.4 (3)
Cu1—O1—C1—O2	−19.3 (3)	C9—C10—C11—C13	177.1 (2)
Cu1—O1—C1—C2	157.31 (13)	C10—C11—C12—N1	0.7 (3)
O1—C1—C2—C3	164.19 (19)	C13—C11—C12—N1	−177.08 (18)
O1—C1—C2—C7	−20.9 (3)	C10—C11—C13—O3	−174.0 (2)
O2—C1—C2—C3	−19.0 (3)	C10—C11—C13—N2	4.7 (3)
O2—C1—C2—C7	156.0 (2)	C12—C11—C13—O3	3.6 (3)
C1—C2—C7—C6	−171.9 (2)	C12—C11—C13—N2	−177.76 (19)
C3—C2—C7—C6	3.0 (3)	C11—C12—N1—Cu1	179.75 (15)
C4—C3—C2—C1	173.1 (2)	C11—C12—N1—C8	−0.6 (3)

Symmetry codes: (i) −*x*, −*y*, −*z*+1.

### Hydrogen-bond geometry (Å, °)

**Table:**

Cg is the centroid of the N1/C8–C12 pyridine ring.

**Table:**

*D*—H···*A*	*D*—H	H···*A*	*D*···*A*	*D*—H···*A*
N2—H21···O2^ii^	0.85 (3)	2.06 (3)	2.831 (2)	151 (3)
N2—H22···O3^iii^	0.88 (3)	2.03 (3)	2.893 (3)	166 (3)
O4—H41···O2^i^	0.87 (4)	1.86 (4)	2.718 (2)	167 (4)
O4—H42···O3^iv^	0.78 (4)	2.17 (4)	2.911 (2)	159 (4)
C6—H6···O2^v^	0.95	2.43	3.377 (3)	172
C4—H4···Cg^vi^	0.95	2.63	3.581 (3)	176

Symmetry codes: (ii) −*x*, −*y*, −*z*+2; (iii) −*x*+1, −*y*, −*z*+2; (i) −*x*, −*y*, −*z*+1; (iv) −*x*+1, −*y*, −*z*+1; (v) *x*+1, *y*, *z*; (vi) *x*, *y*+1, *z*.
